# Neurocysticercosis-Induced Monoparesis: A Case Report of a Rare Neurological Manifestation

**DOI:** 10.7759/cureus.62587

**Published:** 2024-06-18

**Authors:** Vaibhav, Rohit Sharma, H.K. Aggarwal, Shaveta Dahiya, Vipul Kaliraman

**Affiliations:** 1 Department of Neurology, Pandit Bhagwat Dayal Sharma University of Health Sciences, Rohtak, IND; 2 Department of Medicine, Pandit Bhagwat Dayal Sharma University of Health Sciences, Rohtak, IND; 3 Department of Medicine, Maulana Azad Medical College, New Delhi, IND

**Keywords:** ncc, taenia solium, magnetic resonance imaging, cysticercosis, monoparesis, neurocysticercosis

## Abstract

Neurocysticercosis (NCC), a disease caused by the larval form of the pork tapeworm *Taenia solium*, is a common cause of acquired epilepsy globally, especially in areas with poor sanitation. While seizures and headaches are common manifestations, cases of NCC leading to monoparesis are exceedingly rare. Here, we describe a distinctive case of a 42-year-old male who developed sudden weakness and spasms in his left hand without prior injury or other systemic symptoms. Magnetic resonance imaging (MRI) of the brain revealed a single cystic lesion in the right frontoparietal lobe indicative of NCC, which was the cause of his pure motor monoparesis (PMM), without any sensory loss. Treatment with dexamethasone and albendazole substantially improved his motor abilities, highlighting the necessity of considering NCC in differential diagnoses for monoparesis, particularly in endemic areas. This case adds a unique perspective to the clinical spectrum of NCC, highlighting the critical role of prompt and accurate diagnosis followed by appropriate treatment in achieving favorable outcomes.

## Introduction

Central nervous system involvement is not uncommon in individuals afflicted with cysticercosis, an ailment induced by the larval stage of the pork tapeworm, *Taenia solium*, denoted as neurocysticercosis (NCC) [1]. Neurocysticercosis continues to be the prevailing helminthic neurological infection particularly rampant in Latin America, South and Southeast Asia, and sub-Saharan Africa, demonstrating a heightened prevalence and hyperendemicity in these regions [2]. The estimated global population affected by symptomatic and asymptomatic neurocysticercosis ranges between 2.56 and 8.30 million individuals [2].

The clinical manifestations of NCC hinge on the cyst's location, the number of lesions, and the host's inflammatory response [1]. In cases involving intraparenchymal lesions, presentations commonly manifest as seizures and headaches, with focal neurological signs being a less frequent occurrence [3]. Extraparenchymal neurocysticercosis is associated with various complications, including mass effects, hydrocephalus, chronic arachnoiditis, increased intracranial pressure (e.g., headache, nausea, and vomiting), and vasculitis [4]. Pure motor monoparesis (PMM), specifically, is an exceedingly rare presentation within this context [1,4]. This report details the case of an adult presenting with a solitary intraparenchymal neurocysticercosis lesion, characterized by pure motor monoparesis in the left hand.

## Case presentation

A 42-year-old male presented to the emergency room with the chief complaint of spasms and weakness in the left hand persisting for the last 72 hours. The patient reported tremors in the left hand upon attempting to move the fingers, and he was unable to make a fist or extend the fingers of the left hand. Additionally, the patient noted a tingling sensation over the left eyebrow for the same duration.

The patient's blood pressure was 116/80 mmHg, pulse rate was 65 bpm, and oxygen saturation was 99% on room air, and the patient had no abnormalities on respiratory, abdominal, and cardiovascular system examinations. Upon neurological examination, the motor strength of the small muscles of the left hand was 3/5, and the grip strength in the left hand was poor, while the motor strength in the rest of the body remained 5/5. Spasticity was noted in all movements of the left-hand joints, with the inverted supinator reflex registering at 3+. Other deep tendon reflexes were graded at 2+. The patient had normal higher mental functions with an IQ of 90, and no sensory or cranial nerve deficit was observed.

**Figure 1:**
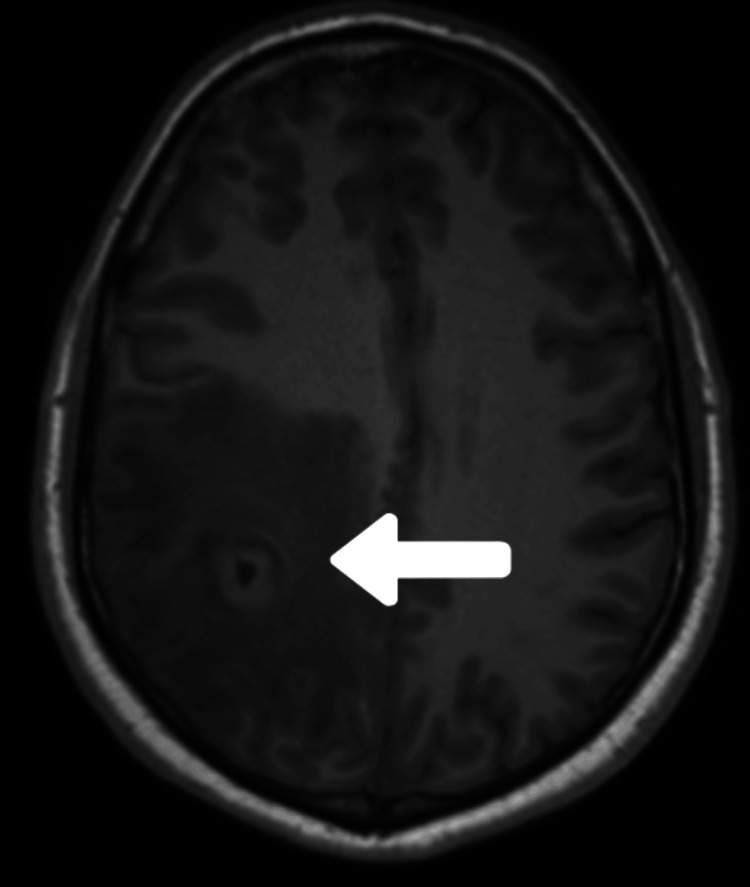
T1-weighted MRI demonstrating vesicular cysticercus Axial T1-weighted MRI demonstrates a single vesicular stage of cysticercus (white arrow) in the right frontoparietal lobe. MRI: magnetic resonance imaging

**Figure 2:**
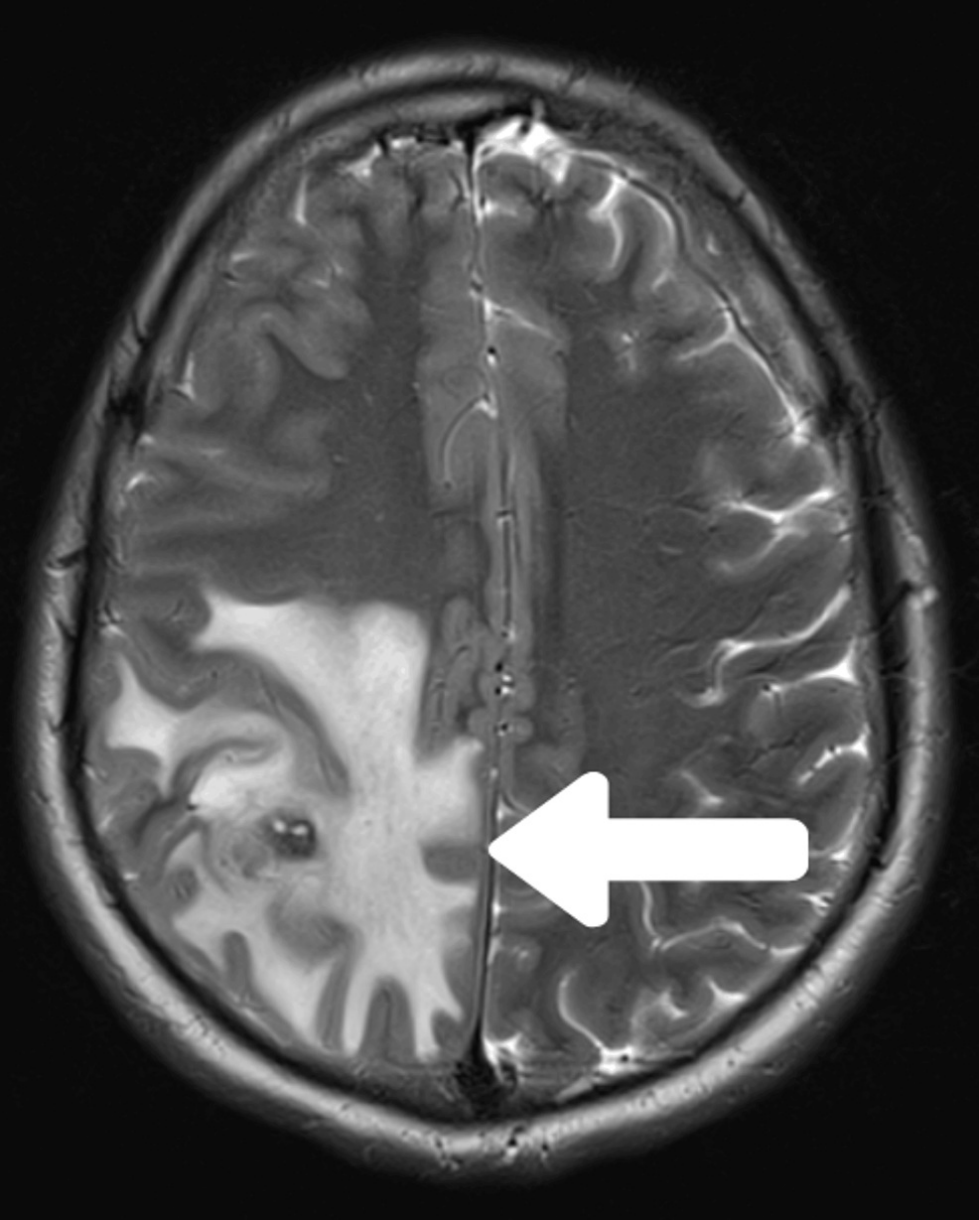
T2-weighted MRI sequence T2-weighted MRI sequence showing hyperintense core with perilesional edema (white arrow). MRI: magnetic resonance imaging

Contrast-enhanced magnetic resonance imaging (CEMRI) of the brain and cervical spine revealed a well-defined, 6.9 × 6.8 mm, round area of abnormal signal intensity located at the juxtacortical region of the right frontoparietal lobe, involving the precentral gyrus. The lesion displayed a hypointense core with an isointense periphery on T1-weighted and fluid-attenuated inversion recovery (FLAIR) sequences (Figure 1). The lesion showed a hyperintense core on the T2-weighted sequence (Figure 2). Perilesional edema was evident. Extraparenchymal lesions were not detected. Magnetic resonance spectroscopy (MRS) showed an increase in lactate and choline peaks and a decrease in N-acetylaspartate (NAA) peak. The enzyme-linked immunosorbent assay (ELISA) for cysticercosis IgM antibody in serum yielded a positive result.

The definitive diagnosis, in this case, was made as neurocysticercosis with an intraparenchymal cystic lesion, resulting in a focal neurological deficit characterized by pure motor monoparesis. As there was no evidence of ischemia on diffusion-weighted imaging (DWI) and apparent diffusion coefficient (ADC) map and no evidence of bleeding on fast field echo (FFE) sequence in MRI, stroke was excluded from consideration. The patient was initiated on dexamethasone at 6 mg daily for a duration of five days, followed by oral albendazole at a dosage of 800 mg daily for the next 14 days. Subsequent follow-up visits indicated improvement in the patient's neurological deficit.

## Discussion

In this report, we present a case of pure motor monoparesis attributed to parenchymal neurocysticercosis, a condition exceptionally rare and frequently subject to misdiagnosis or oversight. Pure motor monoparesis (PMM) constitutes a heterogeneous syndrome arising from diverse pathological processes impacting the pyramidal pathway at both supratentorial and infratentorial levels [5]. Common etiologies of PMM encompass ischemic stroke, hemorrhage, occluded carotid artery, and infrequently, neurocysticercosis, as in this case [5]. 

The patient in this case manifested spasticity and weakness in the left hand, with signs of hyperreflexia, indicative of an upper motor neuron (UMN) lesion. Subsequent confirmation through MRI identified a lesion in the right frontoparietal lobe, categorized as intraparenchymal, more prevalent than extraparenchymal lesions [1,6,7]. Intraparenchymal lesions typically localize at the gray-white matter junction, postulated to stem from larvae deposition in terminal small vessels within this region [6]. Lesion numbers may vary, contributing to the spectrum of clinical presentations. Contrast-enhanced MRI is optimal for visualizing acute symptomatic lesions, while non-contrast CT is preferred for chronic lesions, showcasing hyperdense calcifications [4,6]. The combination of clinical and radiological findings yields a diagnostic sensitivity and specificity of up to 99.5% and 98.9%, respectively, particularly for a single enhanced lesion [2,8].

Clinical manifestations of neurocysticercosis (NCC) are intricately linked to individual variations in lesion number, size, topography, and the host's immune response severity. Consequently, the spectrum of disorders associated with neurocysticercosis ranges from relatively uncomplicated presentations, such as the simple monoparesis observed in this patient, to more complex and potentially fatal conditions [7]. Parenchymal NCC predominates over extraparenchymal NCC in frequency, although the latter carries a heightened risk of mortality [9,10]. Seizures represent the most prevalent manifestation of intraparenchymal NCC, frequently presenting as focal seizures that may progress to secondary generalization [7]. Interestingly, in the Indian subcontinent, the disease spectrum appears to be confined predominantly to single intraparenchymal lesions [4].

Calcified NCC lesions are commonly identified on CT scans in adults with seizures in endemic regions [11]. Cysticercotic encephalitis, a rare presentation of parenchymal NCC, is characterized by elevated intracranial pressure resulting from diffuse cerebral edema triggered by an intense immune response to numerous parenchymal cysts [12]. Many parenchymal NCC cases, however, remain asymptomatic, with the parasites naturally dying without intervention, often incidentally detected through radiographic imaging [12]. The most common extraparenchymal presentation is intraventricular cysticercosis, followed by subarachnoid cysticercosis [4,13]. Intraventricular lesions may manifest in various ventricles, appearing as cysts attached to the choroid plexus or free-floating cysts capable of migrating between ventricles [4,13].

Mechanical obstruction in cerebrospinal fluid (CSF) outflow through ventricles may result from cysts growing larger than 1-2 cm or secondary to inflammation, leading to intracranial hypertension, focal neurological deficits, or brainstem compression (particularly in the case of fourth ventricle cysts) [4,7,13,14]. Mobile cysts in the third and fourth ventricles can induce intermittent obstruction, giving rise to episodes of sudden-onset symptoms associated with head movements, known as Bruns syndrome [15].

Subarachnoid NCC cysts predominantly develop in Sylvian fissures, basilar cisterns, interhemispheric spaces, and the spinal cord, often affecting multiple sites [4,16]. Cysts in these locations typically lack a scolex and are classified as the racemose form of cysticerci, which are larger than the cellulosae form [17].

Hydrocephalus stands as the most common complication of subarachnoid cysticercosis, possibly arising from chronic arachnoiditis and subsequent fibrosis of arachnoid villi or occlusion of the foramina of Luschka and Magendie by thickened leptomeninges [17]. While spinal cord involvement in cysticercosis is rare, it predominantly occurs in the subarachnoid space and may present with symptoms indicative of spinal cord compression [18].

Ocular cysticercosis represents another rare presentation, often manifesting as proptosis with restricted ocular movements. Other ocular manifestations include subconjunctival cysts, subretinal cysts, papilledema, atypical optic neuritis, lid nodules, and intraretinal cysts [19].

The management of neurocysticercosis is contingent upon the clinical presentation and the characteristics, as well as the location, of lesions within the central nervous system. For patients exhibiting viable intraparenchymal neurocysticercosis with 1-2 viable parenchymal cysticerci, albendazole monotherapy (15 mg/kg/day in two daily doses up to 1,200 mg/day) is the recommended treatment. In cases where more than two viable parenchymal cysticerci are present, dual antihelminthic therapy comprising albendazole (15 mg/kg/day in two daily doses up to 1,200 mg/day) and praziquantel (50 mg/kg/day in three daily doses) is advocated. Corticosteroid therapy is advised prior to antiparasitic therapy for all patients, irrespective of the vitality or degeneration status of intraparenchymal neurocysticercosis lesions. The use of corticosteroids is associated with fewer incidences of seizures as it reduces the inflammation associated with the use of antiparasitic drugs. Commonly used regimens include prednisone (1 mg/kg/day) or dexamethasone (0.1 mg/kg/day). Additionally, antiepileptic drugs are recommended for individuals presenting with seizures. Existing literature describes the use of phenytoin or carbamazepine. However, newer drugs, such as levetiracetam, likely have fewer drug-drug interactions. The patient in this case was treated along the same lines.

For patients with degenerating intraparenchymal neurocysticercosis, albendazole monotherapy is the preferred treatment. Periodic MRI assessments, repeated every six months, are mandated until the resolution of cystic lesions in both viable and degenerating intraparenchymal neurocysticercosis.

In instances of calcified parenchymal lesions, symptomatic therapy is advocated instead of antiparasitic drugs. Surgical removal of seizure foci is suggested for cases with refractory epilepsy accompanied by calcified parenchymal lesions. For cysticerci located in the lateral or third ventricle, minimally invasive neuroendoscopy is recommended over surgical or medical approaches. Conversely, for cysticerci located in the fourth ventricle, surgical removal takes precedence over medical therapy or shunt surgery.

In cases of adherent intraventricular neurocysticercosis or subarachnoid neurocysticercosis leading to hydrocephalus, shunt surgery is the recommended course of action. Corticosteroids are advised in the perioperative period to mitigate brain edema. Uncomplicated subarachnoid neurocysticercosis is recommended to be initially treated with corticosteroids, followed by antiparasitic therapy. Spinal neurocysticercosis necessitates a combined medical and surgical approach, while intraocular neurocysticercosis is preferably managed through surgical removal rather than antiparasitic drugs. In cases of pregnancy, anthelmintic therapy should be deferred until after pregnancy, and due consideration should be given to the teratogenicity of antiepileptic drugs [2,10].

## Conclusions

Neurocysticercosis remains a largely neglected condition, despite being a major cause of neurological disorders in regions with poor sanitation. To our knowledge, pure motor monoparesis as a complication of neurocysticercosis has been documented only once in the medical literature. This condition often remains undiagnosed or is frequently misdiagnosed. The patient in this case presented with a UMN lesion at the age of 42, which usually indicates a diagnosis of cerebrovascular events, specifically stroke. However, while considering differential diagnoses for this clinical presentation, neurocysticercosis should also be considered especially in endemic areas.

## References

[1] Coyle CM (2019). Neurocysticerosis: an individualized approach. Infect Dis Clin North Am.

[2] World Health Organization (2021). WHO guidelines on management of Taenia solium neurocysticercosis. https://iris.who.int/bitstream/handle/10665/344802/9789240032231-eng.pdf.

[3] Del Brutto OH (2014). Neurocysticercosis. Handb Clin Neurol.

[4] Garcia HH, Gonzalez AE, Gilman RH (2020). Taenia solium cysticercosis and its impact in neurological disease. Clin Microbiol Rev.

[5] Paciaroni M, Caso V, Milia P (2005). Isolated monoparesis following stroke. J Neurol Neurosurg Psychiatry.

[6] Lerner A, Shiroishi MS, Zee CS, Law M, Go JL (2012). Imaging of neurocysticercosis. Neuroimaging Clin N Am.

[7] Garcia HH, Nash TE, Del Brutto OH (2014). Clinical symptoms, diagnosis, and treatment of neurocysticercosis. Lancet Neurol.

[8] Morgado C, Gomes LB, de Campos JG (1994). [Neurocysticercosis. An imaging analysis of 35 cases]. Acta Med Port.

[9] Abanto J, Blanco D, Saavedra H (2021). Mortality in parenchymal and subarachnoid neurocysticercosis. Am J Trop Med Hyg.

[10] White AC Jr, Coyle CM, Rajshekhar V (2018). Diagnosis and treatment of neurocysticercosis: 2017 clinical practice guidelines by the Infectious Diseases Society of America (IDSA) and the American Society of Tropical Medicine and Hygiene (ASTMH). Clin Infect Dis.

[11] Nash TE, Pretell EJ, Lescano AG, Bustos JA, Gilman RH, Gonzalez AE, Garcia HH (2008). Perilesional brain oedema and seizure activity in patients with calcified neurocysticercosis: a prospective cohort and nested case-control study. Lancet Neurol.

[12] Takayanagui OM, Odashima NS (2006). Clinical aspects of neurocysticercosis. Parasitol Int.

[13] Nash TE, Ware JM, Mahanty S (2018). Intraventricular neurocysticercosis: experience and long-term outcome from a tertiary referral center in the United States. Am J Trop Med Hyg.

[14] García HH, Gonzalez AE, Evans CA, Gilman RH (2003). Taenia solium cysticercosis. Lancet.

[15] Torres-Corzo J, Rodriguez-della Vecchia R, Rangel-Castilla L (2006). Bruns syndrome caused by intraventricular neurocysticercosis treated using flexible endoscopy. J Neurosurg.

[16] Nash TE, O'Connell EM, Hammoud DA, Wetzler L, Ware JM, Mahanty S (2020). Natural history of treated subarachnoid neurocysticercosis. Am J Trop Med Hyg.

[17] Fleury A, Carrillo-Mezo R, Flisser A, Sciutto E, Corona T (2011). Subarachnoid basal neurocysticercosis: a focus on the most severe form of the disease. Expert Rev Anti Infect Ther.

[18] Callacondo D, Garcia HH, Gonzales I, Escalante D, Nash TE (2012). High frequency of spinal involvement in patients with basal subarachnoid neurocysticercosis. Neurology.

[19] Pushker N, Bajaj MS, Chandra M, Neena Neena (2001). Ocular and orbital cysticercosis. Acta Ophthalmol Scand.

